# Personalized, parcel‐guided rTMS for the treatment of major depressive disorder: Safety and proof of concept

**DOI:** 10.1002/brb3.3268

**Published:** 2023-10-05

**Authors:** Si Jie Tang, Jonas Holle, Nicholas B. Dadario, Olivia Lesslar, Charles Teo, Mark Ryan, Michael Sughrue, Jacky T. Yeung

**Affiliations:** ^1^ School of Medicine University of California Davis Medical Center Sacramento California USA; ^2^ Cingulum Health Sydney Australia; ^3^ Robert Wood Johnson Medical School Rutgers University New Brunswick New Jersey USA; ^4^ Department of Neurosurgery Yale University School of Medicine New Haven Connecticut USA

**Keywords:** connectomics, functional MRI, individualized, major depressive disorder, rTMS

## Abstract

**Background:**

Not all patients with major depressive disorder (MDD) benefit from the US Food and Drug Administration‐approved use of repetitive transcranial magnetic stimulation (rTMS) at the dorsolateral prefrontal cortex. We may be undertreating depression with this one‐size‐fits‐all rTMS strategy.

**Methods:**

We present a retrospective review of targeted and connectome‐guided rTMS in 26 patients from Cingulum Health from 2020 to 2023 with MDD or MDD with associated symptoms. rTMS was conducted by identifying multiple cortical targets based on anomalies in individual functional connectivity networks as determined by machine learning connectomic software. Quality of life assessed by the EuroQol (EQ‐5D) score and depression symptoms assessed by the Beck Depression Inventory (BDI) were administered prior to treatment, directly after, and at a follow‐up consultation.

**Results:**

Of the 26 patients treated with rTMS, 16 (62%) attained remission after treatment. Of the 19 patients who completed follow‐up assessments after an average interval of 2.6 months, 11 (58%) responded to treatment and 13 (68%) showed significant remission. Between patients classified with or without treatment‐resistant depression, there was no difference in BDI improvement. Additionally, there was significant improvement in quality of life after treatment and during follow‐up compared to baseline.

**Limitations:**

This review is retrospective in nature, so there is no control group to assess the placebo effect on patient outcomes.

**Conclusion:**

The personalized, connectome‐guided approach of rTMS is safe and may be effective for depression. This personalized rTMS treatment allows for co‐treatment of multiple disorders, such as the comorbidity of depression and anxiety.

## Graphical abstract

The personalized, agile approach of rTMS is safe and may have improved efficacy in treating patients with MDD.

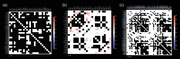

## 1 INTRODUCTION

Major depressive disorder (MDD) is a highly heterogeneous disorder characterized by multiple specifiers based on comorbidities such as phobias, posttraumatic stress disorder, and generalized anxiety disorder (GAD) (Hasin et al., 2018). The constellation of symptoms required to diagnose MDD is not anatomically based despite much previous work attempting to relate depression pathophysiology with isolated cortical regions. More recently, improvements in neuroimaging technologies and our understanding of the brain connectome have provided significant insight into depression and suggest an association with abnormal structural and functional connectivity between various neuroanatomic substrates and large‐scale brain networks (Young, Dadario, et al., 2023). In line with these observations, functional magnetic resonance imaging (fMRI)‐derived connectomic features can be utilized to identify unique MDD “biotypes” with distinctly clinical symptom profiles solely according to distinctively dysfunctional connectivity patterns (Drysdale et al., 2017; Fu et al., 2019; Liang et al., 2020). Incorporating information on individual depression connectomes may also be useful for therapeutic interventions that attempt to modulate various related networks.

Repetitive transcranial magnetic stimulation (rTMS) has emerged as an important therapy for MDD when medication alone is not effective. TMS is a noninvasive device that delivers pulsed magnetic fields to induce an electric field throughout the circuitry of the cerebral cortex. In 2008, the US Food and Drug Administration (FDA) approved the use of rTMS at the dorsal lateral prefrontal cortex (dlPFC) to treat medically resistant MDD (Cohen et al., 2022). One advancement over the years is the use of intermittent theta‐burst stimulation (iTBS) pulses, which was demonstrated to have a 40% response to treatment and better long‐term effects than the traditional high‐frequency rTMS (Cheng et al., 2021; C.‐T. Li et al., 2014). Nonetheless, not all patients with MDD respond well to rTMS, and success is known to vary. One significant reason for various treatment responses is likely due to the fact that the dlPFC is a highly heterogeneous region that can be functionally segregated into many unique spots with various network associations (Glasser et al., 2016). For instance, the authors of the Human Connectome Project (HCP) divided it into 13 unique regions of interest (ROI), and therefore it is not entirely surprising that small differences in the target location in the dlPFC can produce varying responses (Rosen et al., 2021). Although the dlPFC and its part in the Default Mode Network (DMN) have been well studied in relation to depression, numerous other areas of the DMN and other networks including the salience and central executive network (CEN) are also implicated (Dutta et al., 2014; Kaiser et al., 2015; J. Li et al., 2021). Given the vast number of networks and regions, targeting only the dlPFC may mean that we are undertreating patients with depression.

The one‐size‐fits‐all method of the current TMS treatment does not consider the multi‐network dysfunction of MDD nor does it address the various biotypes of MDD. However, the challenge of TMS, and perhaps a possible reason for the low success rates, is the non‐optimized targeting of brain regions (Luber et al., 2017). To treat patients with MDD who have failed standard treatments, a more personalized approach must be developed to address the heterogeneity of MDD. The personalized approach to rTMS treatment of MDD has been previously published in an open‐label proof‐of‐concept study in which rTMS targets were determined by fMRI and then coupled with a type of cognitive behavioral therapy known as self‐system therapy (Neacsiu et al., 2018; Sathappan et al., 2019). The results from that study showed evidence of efficacy in all five participants, thereby encouraging the use of personalized rTMS. Importantly, they found a region in the right dlPFC that was discriminately more active for patients with MDD and generalized anxiety comorbidities than patients with MDD without anxiety, and previous studies from this group have found success in targeting this area in patients with comorbid anxiety and depression (Mantovani et al., 2013; Neacsiu et al., 2018). Given how symptoms and connectivity of MDD vastly affect treatment outcome, it is difficult for one method of dlPFC‐based TMS to be a catch‐all for patients with depression. A more recent study also utilized a personalized approach to identify the dlPFC of the DMN based on the patient's resting‐state fMRI (rsfMRI) before using iTBS to decrease the functional connectivity between the DMN and the salience network (Singh et al., 2020).

Our novel use of machine learning and parcel‐guided rTMS utilizes an individualized, data‐driven approach to identify multiple regions based on the cortical functional connectivity of a patient's brain. We hypothesized that this parcel‐guided and personalized rTMS treatment for depression is safe and may be more efficacious and long‐lasting than previous methods.

## 2 MATERIALS AND METHODS

### 2.1 Subjects

We conducted a retrospective analysis of patients who were treated for depression at Cingulum Health (Sydney, Australia) from January 2020 to April 2023. Patients were recruited to the study if they were at least 16 years of age and diagnosed with MDD or MDD with added specifiers such as anxious distress or melancholic features based on the Diagnostic and Statistical Manual of Mental Procedures Fifth Edition Patients with other co‐morbid psychiatric diagnoses are noted in the Supporting Information. A history of previous rTMS or other brain stimulation was not an exclusion criterion for our study. Patients were excluded if they were unable to undergo functional imaging due to reasons such as claustrophobia or unable to participate in rTMS due to contraindications such as epilepsy. Prior to treatment, a psychiatrist or a qualified primary care clinician must have diagnosed the patient with MDD. The nature and potential risks of rTMS treatment were explained to all patients before they gave informed consent. The consensus recommendations given for the clinical application of rTMS for the treatment of MDD by the National Network of Depression Centers rTMS Task Group and the American Psychiatric Association Council on Research Task Force on Novel Biomarkers and Treatments were referenced through our selection of subjects (McClintock et al., 2018). Seven of our 26 patients fit these guidelines and could be considered to have treatment‐resistant depression (TRD) since they have failed two or more antidepressants. We acknowledged that some patients do not fit the patient eligibility for traditional rTMS treatment and may have a preference against medication such that rTMS served as a preferred alternative to medication.

All patients were screened carefully for any contraindications to assess whether they were candidates for TMS. The predominant contraindication was epilepsy as there is already a known seizure risk associated with TMS in healthy patients that increases significantly in patients with epilepsy and other conditions to a lesser degree (Rossi et al., 2021). Hearing protection was offered to all patients but was optional.

Patient data were analyzed retrospectively. This study was approved by the Human Research Ethics Committee of the South Eastern Sydney Local Health District (2022/ETH00139).

### 2.2 Psychological evaluations

EuroQol (EQ‐5D) (version EQ‐5D‐5L) and the Beck Depression Inventory (BDI) (version BDI‐II) questionnaires were submitted by patients prior to therapy on the last day of treatment and during a follow‐up consultation after a minimum interval of 1 month. We defined response and remission based on the guidelines created by Riedel et al. (2010), where a decrease of 47% or more for the BDI baseline score corresponded to a response in treatment and a BDI cut‐off of ≤12 corresponded to remission (Riedel et al., 2010).

### 2.3 Non‐contrast T1 and resting‐state functional MRI acquisition

rsfMRI and non‐contrast T1‐weighted images were collected from patients 1 week prior to rTMS using a Phillips 3T Achieva. The rsfMRI was acquired as a T2‐star echo‐planar imaging sequence with 3 × 3 × 3‐mm voxels, 128 volumes/run, a TE = 27 ms, a TR = 2.8 s, a field of view –256 mm, a flip angle = 90°, and an 8‐min total run time. For T1‐weighted 3D volume acquisition, 1‐mm slices were obtained with no overlap between slices. The field of view covered the whole head and a 256 × 256 matrix was obtained to achieve isotropic imaging.

### 2.4 Image processing

Resting‐state pre‐processing was performed using Omniscient Infinitome software (Sydney, Australia), which is a cloud‐based software on a Kubernetes's serverless framework written in Python.

### 2.5 Use of machine learning‐based parcellation to create a personalized brain atlas

The Infinitome software was used as previously described (Poologaindran et al., 2022; Yeung et al., 2021; Young, Taylor, et al., 2023). Below are the brief technical summaries involved in image processing.

*Preprocessing*: The rsfMRI images were processed using standard processing steps. First, motion correction was performed on T1 and BOLD images with a rigid body alignment. Slices with excess movement (DVARS > 2 sigma from mean slice) were eliminated. DVARS is defined as the temporal change of the root mean squared of the fMRI voxel. The T1 images were skull stripped with convolution neural net, which was then inverted and aligned to the resting‐state BOLD images using a rigid alignment and then used as a mask to skull strip the rsfMRI image. Next, slice time correction and global intensity normalization were performed. To locally match the rsfMRI and T1 images, gradient distortion correction was performed with the diffeomorphic warping method. The CompCor method was used to calculate high variance confounds. Both high variance and motion confounds were regressed out of the rsfMRI image, and the linear and quadratic signals were detrended (global signal regression cannot be performed with this method). Finally, spatial smoothing was performed with a 4‐mm full width at half maximum Gaussian kernel.

*Correlation and anomaly detection*: The personalized atlas created in previous steps was registered to the T1 image and localized to the gray matter regions, allowing for the extraction of an average BOLD time series from all 377 areas (180 parcellations × 2 hemispheres, plus 17 subcortical structures), which created 142,129 correlations. Parcellations were created using the Human Connectome Project Multi‐Modal Parcellation version 1.0 (HCP) atlas parcellations (Glasser et al., 2016). To determine the normal range of correlations for each functional connectivity pair in the matrix, outlier detection was performed using a tangent space connectivity matrix; the results obtained were compared against the tangent space connectivity transformation of 200 normal subject rsfMRI samples collected from healthy controls from the OpenNeuro (https://openneuro.org/) and SchizConnect (http://schizconnect.org) datasets. To reduce the false discovery rate, abnormal connectivity from rsfMRI was determined as a three‐sigma outlier for that correlation after excluding the highest variance 1/3 of pairs (Poologaindran et al., 2022; Tang et al., 2022; Young, Taylor, et al., 2023).

### 2.6 Method of personalized target section

**FIGURE 1:**
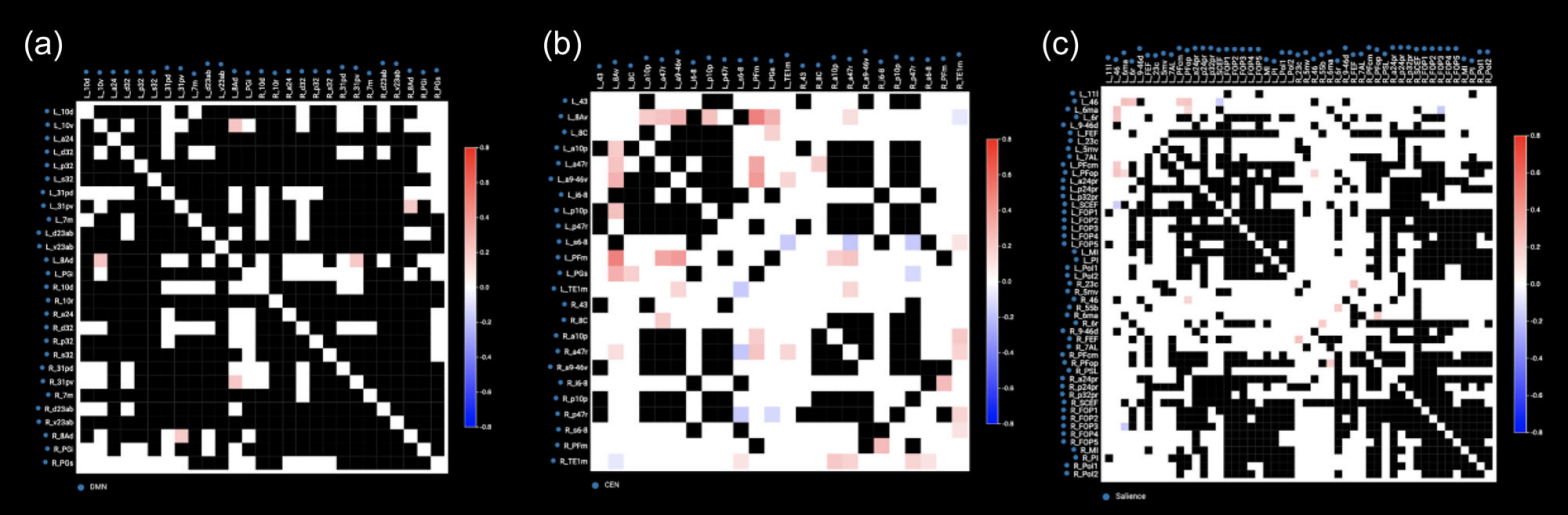
Anomaly detection matrix. (a–c) represent patient 5′s anomaly matrices of their Default Mode Network (DMN), central executive network (CEN), and salience network, respectively. The matrices compare the patient's functional connectivity between each network parcel with the normative functional connectivity in a healthy adult population. Hyperconnected areas are represented in red, hypo‐connected areas are represented in blue, areas within the normal range of correlation are represented in white, and connections represented as black display too much signal noise in healthy populations to determine a normal range. Columns of numerous anomalies within networks implicated in the patient's condition and symptoms become potential targets for stimulation.

Analyses began with the initial hypothesis that functional connectivity abnormalities between parcellations in the DMN, salience, and CEN were the predominant neural basis for depressive symptoms (J. Li et al., 2021; Wise et al., 2017). Anomaly detection matrices were produced for these networks (Figure 1) to detect evidence of abnormal functional connectivity.

No subjective, conscious guidance was given for the targeted therapy for any patient. The selection was made based on the detection of connectivity anomalies outside of three‐sigmas of the normal range from the 200 healthy subject rsfMRI data. The anomaly detection algorithm was used to guide the selection of iTBS or continuous theta‐burst stimulation (cTBS) protocols. In areas that were mostly hyperconnected with other areas, cTBS was chosen to induce cortical depression (Huang et al., 2005; Jung & Lambon Ralph, 2021). For areas that were hypo‐connected to other areas, iTBS was performed (Huang et al., 2005).

### 2.7 rTMS treatment

An accelerated theta‐burst stimulation protocol was used as the treatment. The Localite neuronavigation system (Bonn), which tracked the position of patients’ heads, and the TMS coil, provided real‐time feedback on the location of the coil over representations of cortical targets overlaid upon T1 images. The most common number of targets (and the maximum amount of targets) prescribed to patients was three. Targets at a depth of greater than 30 mm from the surface of the head were not selected as they were assumed to be out of the range of the effective field strength of the Magventure Cool‐365 butterfly coil (Alfaretta) (Deng et al., 2013). Targets were stimulated in sequence in a single session lasting ∼15 min. Five image‐guided TBS treatment sessions per day for 5 days with 1‐h gaps between sessions were conducted as described in a previous study (Sonmez et al., 2019). All targets listed for each subject were consecutively stimulated in each treatment session at 80% of the resting motor threshold. iTBS was performed as bursts of three‐pulse 50‐Hz bursts given every 200 ms at 5 Hz for 40 trains, with an inter‐train interval of 6.3 s, for a total of 1200 pulses. cTBS was performed as one train of 600 stimuli applied in three‐pulse 50‐Hz bursts given every 200 ms at 5 Hz for a total of 1800 pulses. All TBS sessions were completed using a Magventure MagPro X100 TMS machine with a butterfly cool coil (Alfaretta).

### 2.8 Statistical analysis

Mixed‐effects analysis with post hoc Dunnett's multiple comparisons test, unpaired *t*‐test, and Mann–Whitney tests were performed using GraphPad Prism 9 as applicable. A *p*‐value of <.05 was considered significant.

## 3 RESULTS

### 3.1 Patient demographics

Twenty‐six patients (Table 1) met our criteria for intervention and were treated with personalized, parcel‐guided rTMS. Notably, one patient had previously undergone electroconvulsive therapy as a treatment for depression. Twenty‐two patients failed two or more drug trials for depression. Seven patients fit the TRD category as they had failed more than two drug trials. The average follow‐up time was 2.6 ± 2.4 months. Seven patients were unable to complete follow‐up assessments.

**TABLE 1:** Patient Demographics.

General information	Patients (N = 26)
Age (years ± SD)	48.0 ± 14.9
Age at onset of depression (years)	33.7 ± 14.2
Duration of depression (years)	14.2 ± 11.1
Number of adequate antidepressant trials (lifetime)	2.5 ± 1.98
Sex	
Male—Number (%)	17 (65.4)
Female—Number (%)	9 (34.6)
Undergone ECT (number (%))	1 (4)
Most common medications^a^ (number (%))	
Sertraline	8 (30.8)
Venlafaxine	4 (15.3)
Quetiapine	3 (11.5)
Duloxetine	2 (7.6)
Clinical description	
BDI baseline score (scale 0–63) (mean ± SD)	25.2 ± 7.7 (Moderate depression)
Last day of treatment	14.5 ± 8.9
Follow‐up	12.1 ± 9.5
EQ‐5D baseline score (scale −1 to 1) (mean ± SD)	0.524 ± 0.223
Last day of treatment	0.735 ± 0.212
Follow‐up	0.717 ± 0.215
Treatment conditions (number (%))	
cTBS	69 (91)
iTBS	7 (9)

Abbreviations: BDI, Beck Depression Inventory; cTBS, continuous theta‐burst stimulation; ECT, electroconvulsive therapy; iTBS, intermittent theta‐burst stimulation.

^a^A medication list for each patient can be found in the Supporting Information.

### 3.2 Common targets for rTMS

**FIGURE 2:**
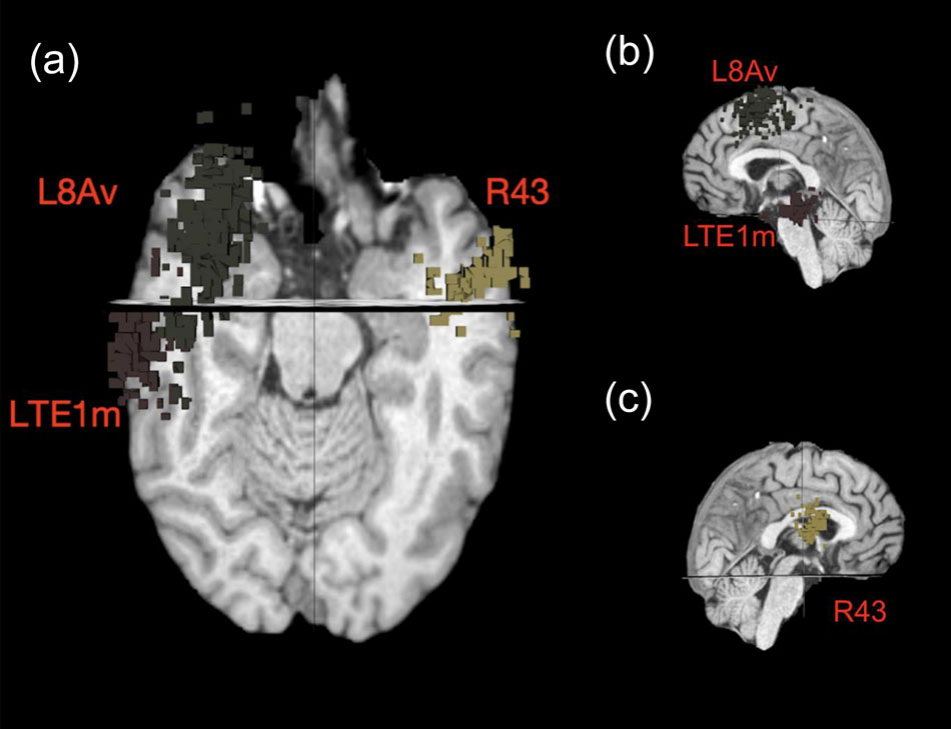
Anatomical locations of repetitive transcranial magnetic stimulation (rTMS) for patient 1. The patient was diagnosed with major depressive disorder (MDD) and generalized anxiety disorder at the age of 43 years. He is currently on sertraline and had failed two antidepressants in the past. T1‐weighted magnetic resonance imaging (MRI). Axial (a), left sagittal (b) and right sagittal (c).

**FIGURE 3:**
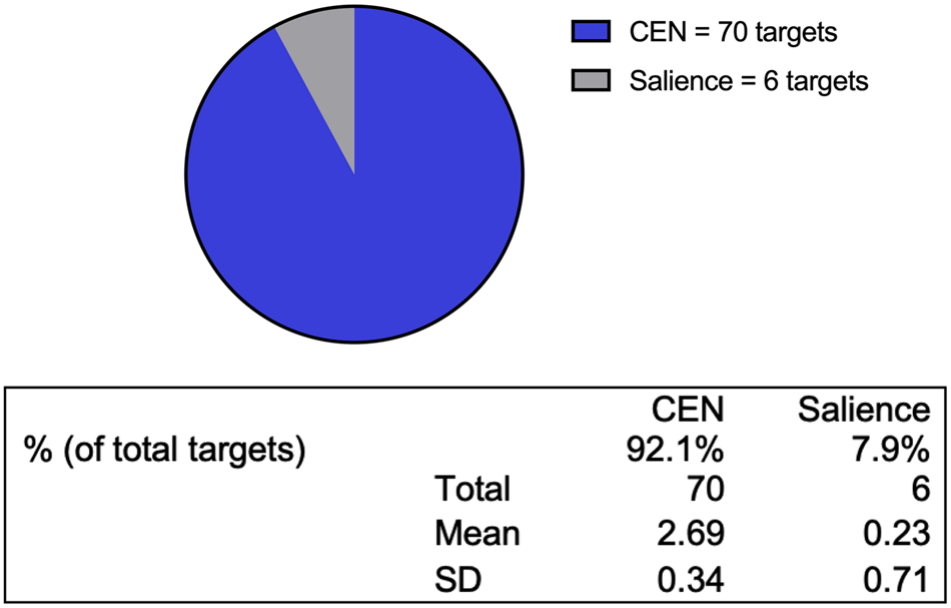
Percentage of targets within the central executive network (CEN) and salience networks.

The targets for rTMS in this cohort of 26 patients are found in Table 2. Targets are labeled based on the parcellations described in Gasser's atlas (Glasser et al., 2016). An example of regions targeted for rTMS is shown for patient 1 in Figure 2. Details of each patient's clinical descriptions are outlined in the Supporting Information. All except for patients 9 and 16 had at least one target within the dlPFC. Out of the 76 total targets in the collective 26‐patient cohort, 66 of those regions (87%) were targeted with cTBS. Out of the 76 total regions, 38 were within the dlPFC (50%). Cumulatively, the targets were most frequently from the CEN (Figure 3). The CEN was targeted in all patients, except for patient 3 who had all three targets within the salience network.

**TABLE 2:** Number of patients with each target location and sequence.

Location (sequence)	Full name	Location	Network	Number of patients with this target (%) total targets = 76
L46 (cTBS)	Left area 46	Brodmann area 46	Salience	3 (3.9)
L46 (iTBS)	Right area 46	Brodmann area 46	Salience	1 (1.3)
L8Av (cTBS)	Left area 8Av	Dorsolateral prefrontal cortex	CEN	17 (22.4)
L8Av (iTBS)	Left area 8Av	Dorsolateral prefrontal cortex	CEN	2 (2.6)
**LPFm (cTBS)**	Left area PFm complex	Inferior parietal cortex	CEN	6 (7.9)
**LPGs (cTBS)**	Left area PGs	Inferior parietal cortex	CEN	13 (17.1)
**LPi (cTBS)**	Para‐insular area	Para‐insular cortex	Salience	1 (1.3)
Ls6‐8 (cTBS)	Left superior 6–8 transition area	Dorsolateral prefrontal cortex	CEN	5 (6.6)
**LTe1m (cTBS)**	Left area TE1 middle	Lateral temporal cortex	CEN	7 (9.2)
**LTe1m (iTBS)**	Left area TE1 middle	Lateral temporal cortex	CEN	1 (1.3)
**R43 (cTBS)**	Right area 43	Brodmann area 43	CEN	2 (2.6)
R46 (iTBS)	Right area 46	Brodmann area 46	Salience	1 (1.3)
**R5mv (cTBS)**	Right area 5 m ventral	Paracentral lobule	Salience	1 (1.3)
R8Av (cTBS)	Right area 8Av	Dorsolateral prefrontal cortex	CEN	2 (2.6)
RIFJp (iTBS)	Right area IFJp	Dorsolateral prefrontal cortex	CEN	1 (1.3)
Rp47r (iTBS)	Right area posterior 47r	Dorsolateral prefrontal cortex	CEN	1 (1.3)
**RPFm (cTBS)**	Right area PFm complex	Inferior parietal cortex	CEN	1 (1.3)
Rs6‐8 (cTBS)	Right superior 6–8 transition area	Dorsolateral prefrontal cortex	CEN	4 (5.3)
**RTe1m (cTBS)**	Right area TE1 middle	Lateral temporal cortex	CEN	7 (9.2)

*Note*: Targets locations are based on parcellations described in Glasser's atlas (Glasser et al., 2016). Targets in bold are outside of the dlPFC.

Abbreviations: CEN, central executive network; cTBS, continuous theta‐burst stimulation; iTBS, intermittent theta‐burst stimulation.

L8Av, a target within the CEN and the dlPFC, was the most common target. It was shared by 19 patients. The most common regions outside of the dlPFC included LTe1m, RTe1m, and LPFm, which were present in eight, eight, and six patients, respectively. These three areas are part of the CEN.

### 3.3 Quality of life and treatment response

**FIGURE 4:**
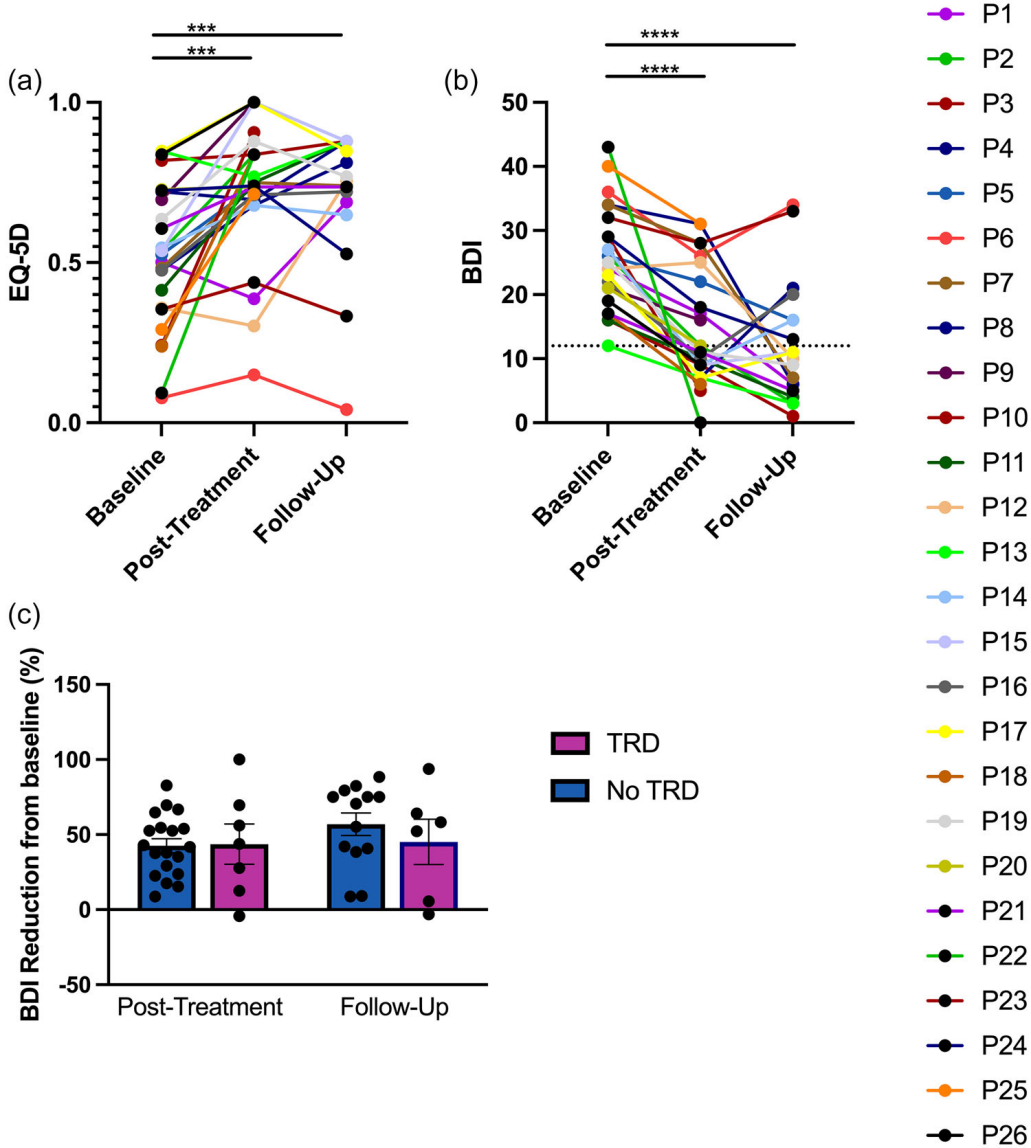
EuroQol (EQ‐5D) and the Beck Depression Inventory (BDI) scores of patients. (a) EQ‐5D (scale −1 to 1) scores of 26 patients at baseline prior to treatment and after treatment. Nineteen patients had follow‐up scores. A mixed‐effects analysis was conducted with Dunnett's multiple comparisons test. There was an improvement after treatment (*p* = .0001, N = 26) and during follow‐up (*p* = .0001, N = 19) compared to baseline. (b) BDI (scale 0–63) scores of 26 patients at baseline prior to treatment and after treatment. Nineteen patients had follow‐up scores. A mixed‐effects analysis was conducted with Dunnett's multiple comparisons test. There was an improvement after treatment (*p* < .0001, N = 26) and during follow‐up (*p* < .0001, N = 19). Dotted line corresponds to BDI remission score. (c) Percentage difference in BDI reduction during post‐treatment and follow‐up for patients who may be considered to have treatment‐resistant depression (TRD) or no TRD. There was no difference in BDI reduction between these two groups during post‐treatment (*p* = .9216, Mann–Whitney test, N = 7–19) and follow‐up (*p* = .4662, Mann–Whitney test, N = 6–13) compared to baseline. *** denote a p‐value < 0.001; **** denotes a p‐value < 0.0001

Patient EQ‐5D (Figure 4a) and BDI (Figure 4b) scores were collected throughout treatment visits. Quality of life was assessed by the EQ‐5D. A mixed‐effects analysis with post hoc Dunnett's multiple comparisons test found an increase in EQ‐5D scores after treatment (*p* = .0001, N = 26) and during follow‐up (*p* = .0001, N = 19) compared to baseline.

Changes in BDI from baseline were also assessed with a mixed‐effects analysis with post hoc Dunnett's multiple comparisons test, revealing an improvement after treatment (*p* < .0001, N = 26) and during follow‐up (*p* < .0001, N = 19). After treatment, eight of 26 patients (31%) responded to treatment, and 16 patients (62%) achieved remission. However, during the 3‐month follow‐up, 11 of 19 patients who reported for follow‐up showed response to therapy (58%), and 13 patients (68%) achieved remission. To determine if there was a difference in BDI reduction for patients who may be considered to have TRD or no TRD, the percent reduction in BDI from baseline was compared between the two groups. Baseline scores of the two groups were matched as *a* t‐test found no difference in BDI scores between these two groups at baseline (*p* = .2021, unpaired *t*‐test, N = 7–19). There was no difference in BDI improvement between patients with TRD and those with no TRD (Figure 4c) during post‐treatment (*p* = .9216, Mann–Whitney test, N = 7–19) and follow‐up (*p* = .4662, Mann–Whitney test, N = 6–13).

### 3.4 Comorbidity of MDD and GAD

Eleven patients (patients 1, 7, 13, 15–19, 21, 24, and 25) were diagnosed with both MDD with GAD. The L8Av and LPGs were common targets, which were shared in ten and seven patients, respectively. Four (36%) responded to treatment, and seven (64%) achieved remission directly after treatment. Nine patients returned for follow‐up. During follow‐up, six (67%) responded to treatment, and eight (89%) achieved remission. The most common region of the target, shared by 10 patients, was the L8Av. The second most frequent target was LPGs, which is outside of the dlPFC.

### 3.5 Retreatment efficacy

**FIGURE 5:**
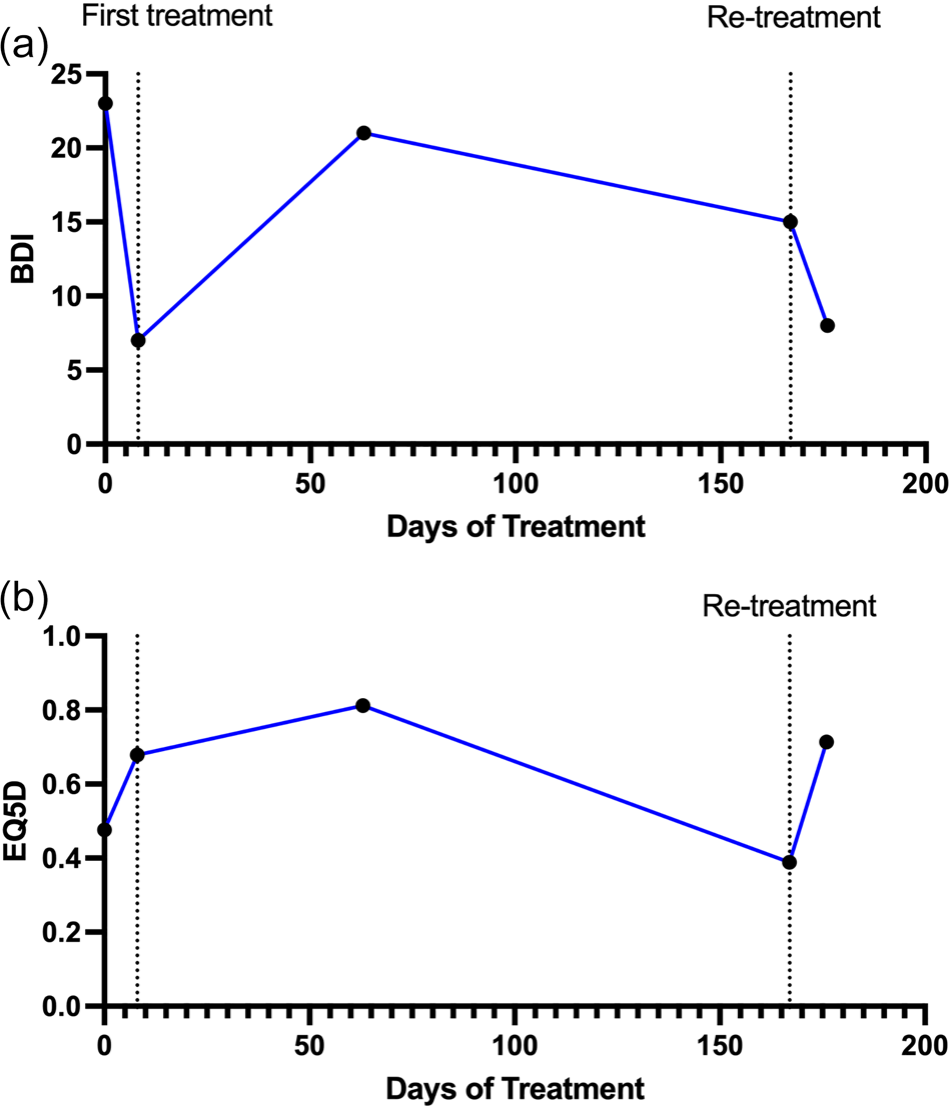
Retreatment efficacy in patient 8. (a) The Beck Depression Inventory (BDI) scores for initial treatment and retreatment. (b) EQ‐5D scores for initial treatment and retreatment. Dotted line at day 167 denotes the first day of retreatment.

Patient 8 was retreated after he/she was no longer at remission during follow‐up. There appeared to be no difference in the magnitude of improvement in BDI and EQ‐5D scores between initial treatment and retreatment (Figure 5a,b).

### 3.6 Safety

Some patients reported some minor side effects during and after treatment (Table 3). To mediate scalp discomfort from stimulation, intensity on sensitive targets was gradually increased from low levels to full percentage to allow acclimatization. All patients were encouraged to maintain hydration during treatment.

**TABLE 3:** Safety of parcel‐guided repetitive transcranial magnetic stimulation (rTMS).

Side effect	Number of patients (%)
Fatigue	10 (38)
Scalp discomfort	4 (15)
Facial twitching	8 (31)
Headache	6 (23)
Alertness	1 (4)

## 4 DISCUSSION

Depression involves dysfunction between several large‐scale brain networks and neuroanatomic substrates and can significantly vary between individuals. Unlike current methods of treating depression with standardized targets across all individuals, a parcel‐guided rTMS approach based on individualized connectomic information may provide a more effective and personalized approach. In this retrospective case series of personalized rTMS treatment for chronic depression, we report that 16 of 26 (62%) patients attained remission at initial follow‐up, and 13 of 19 patients (68%) achieved remission at later follow‐up.

### 4.1 A personalized approach to target the heterogeneity of MDD

MDD is a heterogeneous disorder that can be divided into subtypes based on symptoms and biotypes based on functional connectivity. With 227 different combinations of symptoms, the Research Diagnostic Criteria divides MDD into 11 different subtypes and has shown that there are differences in treatment outcomes based on subtype (Prusoff et al., 1980; van Loo et al., 2012). Two to four biotypes of MDD have been described in the literature through machine learning and resting‐state fMRI (Drysdale et al., 2017). The study found that the combination of connectivity patterns and biotype diagnosis had the highest predictive accuracy of rTMS treatment responsiveness. This diversity may account for the low response rate of patients with depression to the standard FDA‐approved rTMS of the dlPFC.

In this case series, we described a proof‐of‐concept approach to using personalized rTMS to treat chronic depression. By utilizing individual connectomic information to detect unique brain network dysfunction, personalized targets were identified to be modulated with TMS to restore network synchrony. Parcel‐guided and fMRI‐guided rTMS has been previously published as a robust method to provide personalized rTMS treatment with better outcomes than standard rTMS (Cole et al., 2020; Moreno‐Ortega et al., 2020). One recent open‐label rTMS treatment of MDD is the Stanford Accelerated Intelligence Neuromodulation Therapy (SAINT) that utilizes functional connectivity MRI to guide to target the left dlPFC. In 21 patients, SAINT showed a 92% response and 88% remission rate after treatment based on scores of BDI‐II (Cole et al., 2020). It is worth noting that 1 month after SAINT, response and remission rates dropped to 53% and 59%, respectively, suggesting that depressive symptoms gradually return with time. On the other hand, our method showed a much smaller change in BDI scores directly following rTMS treatment, and the most significant improvement in BDI scores occurred at later follow‐up. The SAINT trial and our method suggest that identifying changes in connectivity is as important as monitoring the structure or activation of brain regions (Duman et al., 2019; Menon, 2011). Just like the SAINT trial, we found that there was no difference in BDI and EQ‐5D between initial treatment and retreatment for the one patient who had undergone retreatment. Our study differs from SAINT and other studies in the literature because we identify and treat multiple targets for rTMS through the functional connectivity of individualized brain maps. This approach also allows us to appreciate functional connectivity changes at follow‐up, permitting for the monitoring and modification of treatment if needed.

No subjective, conscious guidance was provided for target selection as it was based on the anomaly matrix detection of hyper‐ or hypoconnectivity. Contrary to other rTMS treatments for depression, many individuals in this study received cTBS rather than iTBS. Unlike the current FDA‐approved rTMS for depression using iTBS to the dlPFC, our study utilized cTBS in 32 of the 38 dlPFC targets due to hyperconnectivity detected in those areas.

Additionally, our study utilizes the novel approach of including the simultaneous targeting of regions that are outside of the dlPFC. Most of our patients had rTMS targets outside of the dlPFC. In previous studies, we have shown that the use of connectome‐guided rTMS can induce neuroplasticity and alter functional connectivity (Yeung et al., 2021; Young, Taylor, et al., 2023). Therefore, it is likely that the effects of rTMS will remain with the patient for the long term, but longer follow‐up is warranted to determine if this holds true.

Furthermore, our method offers potential benefits for patients with psychiatric comorbidities alongside their diagnosis of MDD. In the 11 patients who were diagnosed with depression and anxiety, none of the patients had the exact same three target combinations. Nevertheless, L8Av (within the dlPFC) and LPGs (outside of the dlPFC) were the most common targets shared by these patients. These two areas of the DMN were also common targets in the treatment of anxiety (Young, Taylor, et al., 2023). These results highlight the importance of personalized rTMS, which will account for individualized connectivity differences in treatment regiments. A personalized connectome‐guided approach may facilitate multiple targeting for the co‐treatment of comorbidities. However, reporting of anxiety severity prior to and after rTMS treatment would be informative in corroborating this hypothesis.

### 4.2 Safety

Finally, there were no adverse effects associated with the rTMS treatment. We have previously reported the use of this personalized rTMS method in numerous studies (Dadario et al., 2022; Young, Taylor, et al., 2023; Poologaindran et al., 2022; Yeung et al., 2021), and it has been shown to be safe and able to effectively target multiple ROIs. In the original clinical trials for rTMS, scalp discomfort was identified as the largest contributor to dropping out of the study (O'Reardon et al., 2007). We reported scalp discomfort in 15% of our patients. Fatigue and facial twitching were the most frequent side effect in our study.

### 4.3 Limitations

A limitation to our study is the heterogeneity of our patient population in terms of their history of medication use and MDD diagnosis. Additionally, our trial included individuals with different amounts of drug trial failures. Therefore, the results we observed could be because the depression experienced in our patient sample may not be as severe as other patients in the rTMS clinical trials with more homogenous populations. However, we found no difference in changes in BDI between those patients who were considered to have or did not have TRD. Moreover, all our patients must have been assessed by a physician to have MDD before undergoing rTMS.

Another limitation to our retrospective study is that it is not randomized. The lack of a control group raises the possibility of a placebo effect. Yet, if indeed the results were a placebo effect, the self‐reported scores would have diminished or gone back to baseline during follow‐up because depression shows its placebo response earlier and then gradually reduces with time (F. Li et al., 2019). On the contrary, the patients’ BDI and EQ‐5D scores at follow‐up remain improved (or were improved even more) compared to baseline for all patients except for one who did not respond to treatment. This suggests that the results we observed may not be due to the placebo effect.

This report introduces a novel approach of personalized rTMS treatment for patients with depression. The variety of stimulation targets and protocols combined with the lack of a control group prevent any conclusions from being drawn regarding the efficacy of individual targets and protocols. Rather, this study has sought to document the safety and potential efficacy of a personalized approach to brain stimulation in psychiatric care. Since this approach is still in its seminal stages, additional studies must be performed to verify the true efficacy of this approach through comparison with a sham magnet treatment group. As this study is a case series, it is difficult to draw conclusions on the efficacy of this approach without a control group. Moreover, due to the retrospective nature of the analysis, we recognize that there may be bias in follow‐up scores as seven patients did not complete BDI and EQ‐5D assessments during their follow‐up. Nonetheless, we do see our findings as potential evidence that the personalized and simultaneous targeting of multiple areas with rTMS may be a feasible treatment of MDD. Future controlled studies will be able to elucidate whether this novel approach is effective in treating depression.

## 5 CONCLUSION

A personalized, connectomic approach of rTMS is safe and may be an effective treatment for MDD. This method provides new insight into how simultaneous, multi‐network targeting of rTMS may be efficacious in the treatment of a heterogenous disorder such as MDD.

## AUTHOR CONTRIBUTIONS

**Si Jie Tang**: Formal analysis; visualization; writing—original draft; writing—review and editing. **Jonas Holle**: Data curation; visualization; writing—original draft; writing—review and editing. **Nicholas B. Dadario**: Writing—review and editing. **Olivia Lesslar**: Writing—review and editing. **Charles Teo**: Supervision; writing—review and editing. **Mark Ryan**: Writing—review and editing. **Michael Sughrue**: Supervision; writing—review and editing. **Jacky Yeung**: Conceptualization; investigation; supervision; writing—original draft; Writing—review and editing.

## CONFLICT OF INTEREST STATEMENT

Charles Teo is a founder Omniscient Neurotechnology, and Michael Sughrue is a founder and employee of Omniscient Neurotechnology. Jonas Holle is an employee of Cingulum Health. Jacky Yeung and Olivia Lesslar are consultants of Cingulum Health but are not employees. Si Jie Tang and Nicholas Dadario declare no conflicts of interest. No source of funding was utilized for this work.

### PEER REVIEW

The peer review history for this article is available at https://publons.com/publon/10.1002/brb3.3268.

## Supporting information

Supplement 1. Patient Clinical Details

## Data Availability

Data are available upon request.
