# Supplementary material for: Personalized, parcel‐guided rTMS for the treatment of major depressive disorder: Safety and proof of concept
Source: Brain Behav. 2023 Oct 5;13(11):e3268. doi: 10.1002/brb3.3268 (PMC10636393; doi:10.1002/brb3.3268)
Supplement: Supplementary file 1 — Supplement 1. Patient Clinical Details [file BRB3-13-e3268-s001.docx]

| Patient Number | Age  (years) | Sex | Diagnosis | Age at Diagnosis | Duration of Symptoms (yrs) | Medication | # of Past Anti-depressants | rTMS Targets |
| --- | --- | --- | --- | --- | --- | --- | --- | --- |
| 1 | 59 | M | MDD with generalized anxiety disorder (GAD) | 43 | 16 | Sertraline  (50 mg daily) | 2 | 1. L8Av (iTBS)  **2. LTe1m (iTBS)**  **3. R43 (cTBS)** |
| 2 | 23 | M | MDD | 23 | 4 | None | 0 | 1. L46 (cTBS)#  2. L8Av (cTBS)  **3. LPi (cTBS)#** |
| 3 | 46 | M | MDD with melancholic features | 40 | 26 | Venlafaxine | 3 | 1. R46 (iTBS)# 2. L46 (iTBS)#  **3. R5mv (cTBS)#** |
| 4 | 48 | F | MDD | 22 | 6 | None | 0 | 1. L8Av (cTBS)  **2. LPGs (cTBS)** 3. RP47r (iTBS) |
| 5* | 33 | M | MDD with melancholic features | 47 | 11 | Vortioxetine (10 mg) | 2 | 1. L8Av (cTBS)  **2. LPFm (cTBS)** 3. L46 (cTBS)# |
| 6 | 51 | M | MDD | 20 | 4 | Sertraline  (100 mg) | 4 | 1. Ls6-8 (cTBS) **2. LTe1m (cTBS)**  **3. RTe1m (cTBS)** |
| 7 | 22 | F | MDD with GAD | 19 | 3 | Sertraline, Agomelatine | 2 | 1. L8Av (cTBS)  **2. LPFm (cTBS)**  **3. LTe1m (cTBS)** |
| 8 | 72 | F | Chronic Depression | 68 | 4 | Sertraline, Venlafaxine | 2 | 1.L8Av (cTBS) **2.LPFm (cTBS)** 3.Rs6-8 (cTBS) |
| 9 | 39 | F | MDD | 33 | 6 | Mirtazapine | 2 | **1.LPFm (cTBS)** **2.LTE1m (cTBS)** **3.RTe1m (cTBS)** |
| 10 | 73 | M | MDD | 40 | 30 | Sertraline, Duloxetine | 2 | 1.L8Av (cTBS) **2.LPGs (cTBS)** **3.RTe1m (cTBS)** |
| 11 | 60 | M | Moderate Depression | 15 | 39 | Bromazepam | 0 | 1. L8Av (cTBS)  **2. LPFm (cTBS)**  **3.LTe1m (cTBS)** |
| 12 | 61 | M | MDD with OCD | 30 | 30 | None | 10 | 1. L8Av (cTBS)  **2. LTE1m (cTBS)**  **3.RTe1m (cTBS)** |
| 13 | 55 | M | MDD with GAD | 25 | 30 | Sertraline, Venlafaxine | 2 | 1. L8Av (cTBS)  **2. LPGs (cTBS)**  3.Rs6-8 (CTBS) |
| 14 | 16 | F | MDD with ADHD | 7 | 9 | Sertraline, Lurasidone, Quetiapine, Lisdexamphetamine | 2 | 1. L8Av (cTBS)  2. Ls6-8 (cTBS)  **3. LPGs (cTBS)** |
| 15 | 46 | M | MDD with GAD | 40 | 6 | Sertraline, Diazepam | 2 | 1. L8Av (cTBS)  2. Rs6-8 (cTBS)  **3. LPGs (cTBS)** |
| 16 | 48 | F | MDD with GAD | 30 | 15 | Duloxetine | 2 | **1. LTE1m (cTBS)**  **2. RTE1m (cTBS)**  **3. LPGs (cTBS)** |
| 17 | 45 | M | MDD with GAD | 20 | 28 | Not stated | 3 | 1. L8Av (cTBS)  **2. RTe1m (cTBS)**  3. R43 (cTBS) |
| 18 | 49 | M | MDD with GAD | 35 | 14 | Antidepressant (unspecified) | 2 | 1. L8Av (cTBS)  **2. RPFm (cTBS)**  **3. LPGs (cTBS)** |
| 19 | 51 | M | MDD with GAD | 38 | 13 | Venlafaxine, Sodium valproate | 6 | 1. L8Av (cTBS)  **2. RTE1m (cTBS)**  **3. LPGs (cTBS)** |
| 20 | 33 | M | MDD | 30 | 8 | Escitalopram | 1 | 1. Rs6-8 (cTBS)  **2.LPFm (cTBS)**  **3. LPGs (cTBS)** |
| 21 | 39 | F | MDD with GAD | 34 | 5 | Citalopram | 2 | 1. L8Av (cTBS)  2. Ls6-8 (cTBS)  **3. LPGs (cTBS)** |
| 22 | 52 | M | MDD | 20 | 32 | Not stated | 3 | **1. LTE1m (cTBS)**  2. Ls6-8 (cTBS)  **3. LPGs (cTBS)** |
| 23 | 66 | M | MDD | 54 | 12 | Not stated | 4 | 1. L8Av (cTBS)  2. Ls6-8 (cTBS)  **3. LPGs (cTBS)** |
| 24 | 71 | F | MDD with GAD | 60 | 11 | Lorazepam | 2 | 1. L8Av (iTBS)  2. RIFJp (iTBS) |
| 25 | 46 | F | MDD with GAD; ADHD | 45 | 1 | Quetiapine | 2 | 1. L8Av (cTBS)  2. R8Av (cTBS)  **3. LPGs (cTBS)** |
| 26 | 45 | M | MDD with Addiction | 39 | 6 | Quetiapine | 2 | 1. L46 (cTBS)  2. R8Av (cTBS) |

**Supplement 1. Patient Clinical Details**

Bolded rTMS targets are outside of the dlPFC. All targets are within the CEN unless otherwise stated. #Denotes areas within the Salience Network

*Denotes patient who previously had ECT in 2016
